# TOR and PKA Pathways Synergize at the Level of the Ste11 Transcription Factor to Prevent Mating and Meiosis in Fission Yeast

**DOI:** 10.1371/journal.pone.0011514

**Published:** 2010-07-09

**Authors:** Noelia Valbuena, Sergio Moreno

**Affiliations:** Instituto de Biología Molecular y Celular del Cáncer, Consejo Superior de Investigaciones Científicas (CSIC), Salamanca University, Salamanca, Spain; Duke University Medical Center, United States of America

## Abstract

**Background:**

In the fission yeast *Schizosaccharomyces pombe*, the TOR (target of rapamycin) and PKA (protein kinase A) signaling transduction pathways regulate the expression of genes required for cell growth and sexual differentiation in response to the nutritional environment. Inhibition of Tor2 signaling results in the induction of genes involved in sexual differentiation, and the cells undergo mating and meiosis, even under good nutritional conditions. The same phenotype is observed in mutants in which the PKA pathway is inactive. By contrast, Tor2 overexpression or mutations that hyperactivate PKA signaling impair sexual differentiation, even under poor nutritional conditions. Accordingly, a very important question is to understand the molecular mechanism by which these two pathways coordinately regulate gene expression in response to nutrients.

**Methodology/Principal Findings:**

Here we demonstrate that TOR and PKA pathways operate coordinately to negatively regulate sexual differentiation by inhibiting the nuclear accumulation of the Ste11 transcription factor. However, the Tor2 pathway is unable to block the nuclear localization of Ste11 under good nutritional conditions when the PKA pathway is inactive. Using microarray analyses, we found that both pathways inhibit sexual differentiation by blocking *ste11-*dependent gene expression.

**Conclusions/Significance:**

We conclude that both the PKA and the TOR pathways inhibit Ste11 nuclear accumulation to repress Ste11-dependent gene expression. However, the PKA pathway plays a quantitatively more important role than the TOR pathway in this process.

## Introduction

In the presence of nutrients, the fission yeast *Schizosaccharomyces pombe* reproduces asexually by means of the mitotic cell cycle. Nitrogen depletion promotes cell cycle arrest in G1 and cells undergo sexual differentiation and meiosis to produce four resistant haploid spores that remain dormant until they encounter favorable growth conditions. TOR and PKA are two prominent, evolutionarily conserved signal transduction pathways that couple nitrogen and carbon source availability, respectively, to regulate diverse cell responses that ultimately drive cell growth and proliferation and inhibit sexual differentiation.

TOR is a serine/threonine protein kinase that is structurally and functionally conserved from yeasts to mammals. In mammalian cells, mTOR is a critical player in the TSC1-TSC2/Rheb/mTOR signaling pathway, which regulates cell growth in response to growth factors, nutrients, and energy conditions. TOR is activated by the GTPase Rheb, which is negatively regulated by the TSC1-TSC2 tuberous sclerosis complex [1], [2]. In the cell, TOR forms two types of multiprotein complexes: namely, TOR complex 1 (TORC1) and TOR complex 2 (TORC2) [3], [4]. TORC1 contains Raptor, is sensitive to rapamycin, and mediates the effects on protein synthesis and cell growth. In contrast, TORC2, which contains Rictor, regulates Akt and also affects the actin cytoskeleton [5], [6]. Unlike higher eukaryotes, which contain a single TOR protein, *S. pombe* and *Saccharomyces cerevisiae* have two: Tor1 and Tor2. Contrary to *S. cerevisiae*, the TSC1-TSC2/Rheb/TOR pathway is conserved in *S. pombe*, providing an excellent model to study the TOR pathway. In *S. pombe*, Tor2 forms part of the TORC1 complex and is essential for cell growth and the repression of sexual differentiation, meiosis and sporulation [7], [8], [9], [10], whereas Tor1 is not essential for growth and is included in the TORC2 complex [7], [9], [11].

Cyclic AMP (cAMP) also plays an important role in the regulation of cellular growth and sexual development in *S. pombe*. In several organisms, the level of cAMP changes in accordance to environmental conditions. In eukaryotic cells, the major target of cAMP is Protein Kinase A (PKA or cAMP-dependent protein kinase), which is a serine/threonine protein kinase that responds to glucose and is involved in the regulation of a broad diversity of biological processes, including cell growth and differentiation, nutrient sensing, and physiological stress responses [12], [13], [14]. In fission yeast, the intracellular level of cAMP is kept high during cell growth and division. Conditions unfavorable for growth cause a down-regulation of PKA, exit from the cell cycle, and sexual differentiation. *S. pombe* cells defective in *cgs1*, the gene for the regulatory subunit of cAMP-dependent protein kinase, constitutively generates active PKA and cells scarcely mate and sporulate [15]. In contrast, cells defective in *cyr1,* encoding adenyl cyclase, or in the *pka1* gene, encoding the catalytic subunit of PKA, readily initiate sexual development in rich medium [16], [17]. This response is followed by gross changes in gene expression, indicating that several transcription factors are likely to be regulated by PKA either directly or indirectly.

The fission yeast *ste11* gene encodes an HMG transcription factor, which is responsible for the expression of many genes required for the initiation of sexual development [18], [19]. Upstream from Ste11, another transcription factor, Rst2, induces the expression of *ste11* and is under the regulation of PKA [20]. PKA inhibits Rst2 at two levels: by phosphorylation and by excluding Rst2 from the nucleus [21]. Thus, a decrease in the PKA activity, which naturally results from a lack of environmental nutrients, triggers the activation of Rst2 and, in turn, *ste11* expression and sexual differentiation.

In sum, TOR and PKA are two signal transduction pathways that couple nitrogen and carbon source availability, respectively, to regulate diverse cell responses that ultimately drive cell growth and proliferation and inhibit sexual differentiation. In this study, we further investigated the interplay between the TOR and cAMP signaling pathways, which regulate gene expression mainly in relation to sexual differentiation. Our results indicate that the PKA pathway plays a more important role in repressing *ste11* than the TOR pathway. Both pathways act in synergy but independently of one another, and converge directly at the level of *ste11* expression. This regulatory network probably functions to provide cells with the flexibility necessary to respond to different inputs from two global nutrient sensors.

## Materials and Methods

### Fission yeast strains and media

All *S. pombe* strains used in this study are listed in Table S1 in supplementary material. Standard methods were used for growth, transformation and genetic manipulations [22]. The tagged versions of the *rst2-gfp*, *rst2-HA* and *ste11-gfp* genes were generated by a PCR-based method [23]. Except where specifically indicated, all experiments in liquid culture were carried out in Edinburgh Minimal Medium (EMM) containing the required supplements (except when mentioned), starting with a cell density of 2–4×10^6^ cells/ml, corresponding to the mid-exponential phase of growth. Temperature shift experiments were carried out using a shaking water bath at 32°C.

### Flow cytometry

Approximately 10^7^ cells were collected by centrifugation, fixed in 70% cold ethanol and processed as described [22]. Flow cytometry analysis (FACS) was performed on a Becton-Dickinson FACScan device, using cells stained with propidium iodide. Cell size measurements were accomplished using the forward light scatter (FSC) data of the FACS.

### RNA extraction and Northern blots

Total RNA from cells was isolated by lysis with glass beads in the presence of phenol [22]. 5–10 µg of RNA from each sample was separated on a formaldehyde agarose gel. Northern blot was carried out using Gene ScreenPlus (NEN, Dupont), following manufacturer's instructions. DNA probes were labeled with [^32^P]dCTPs using the Rediprime II Random Prime Labelling System kit (Amersham).

### Protein extraction and Western blots

Protein extracts were obtained using trichloroacetic acid (TCA) extraction, as described previously [24]. For Western blots, 75–100 µg of total protein extract were run on 7,5% SDS-PAGE, transferred to a nitrocellulose filter (Amersham), and probed with mouse anti-HA 12CA5 (Roche Applied Sciences), mouse anti-ste11 (a gift of Dr Olaf Nielsen), mouse anti-GFP (Clontech) and mouse anti-tubulin (a gift of Dr Keith Gull) primary antibodies and, as secondary antibodies, NA 931, anti-mouse IgG, Horseradish Peroxidase (Amersham). Immunoblots were developed using the enhanced chemiluminescence procedure (ECL kit, Amersham). Phos-tag AAL-107 gels (NARD Institute) were used for shifts in the mobility of phosphorylated Rst2 at a final concentration of 100 µM, following the protocol suggested by the manufacturer.

### Epifluorescence microscopy of GFP fusion proteins

Epifluorescence microscopy was carried out using a Zeiss Axioplan 2 fluorescence microscope equipped with an Orca-ER camera (Hamamatsu). To visualize nuclei, Hoechst 33342 was used to stain DNA.

### Microarrays and data analyses

RNA was purified and cleaned using the RNeasy kit (Qiagen). RNA was analyzed with a 2100 Bioanalyzer (Agilent). The wild-type double-strand cDNA synthesis kit (Affymetrix) was used for the synthesis and purification of cDNA, and a Nanodrop spectrophotometer was used for cDNA quantification. cDNA was labeled and hybridized with a *S. pombe* Tiling 1.0FR Array, following the Affymetrix protocol. All the probes included in the arrays were aligned according to the *S. pombe* genome of the Pombe Sanger Institute Project (http://www.sanger.ac.uk/Projects/S_pombe/). To calibrate the sequence-specific probe effect, all data were background-corrected and quantile-normalized. To analyze different levels of transcription, all the normalized data were located in the correct genomic position and were visualized using Artemis (www.sanger.ac.uk/resources/software/artemis/).

## Results

### In *S. pombe*, the TOR and PKA pathways synergize to prevent exit from the cell cycle

In fission yeast, the TOR and PKA pathways drive cell growth and proliferation and inhibit sexual differentiation. Inactivation of Tor2 in rich medium leads to cell cycle exit, G1 arrest and sexual differentiation, meiosis and sporulation [7], [8], [11], [25]. Similarly, cells defective in the catalytic subunit of PKA (*pka1Δ*) or adenylate cyclase (*cyr1Δ)* undergo sexual development, meiosis and sporulation in the absence of nitrogen starvation [26]. In contrast, overexpression of Tor2, or mutants in *cgs1* encoding the regulatory subunit of the PKA, reveal a reduction in mating and sporulation in poor medium [7], [15].

**Figure 1:**
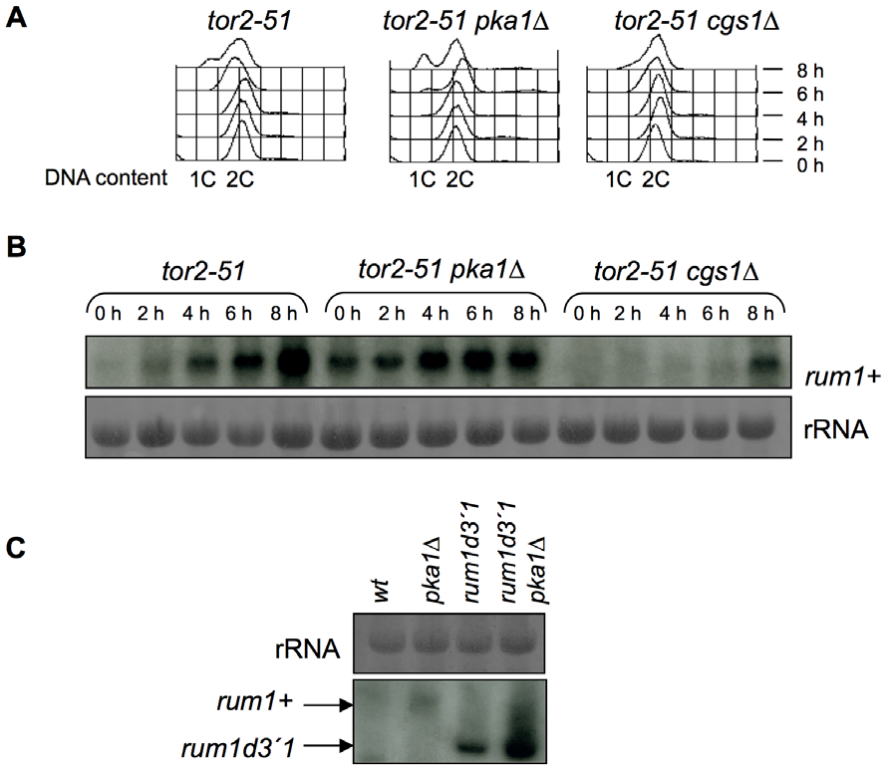
The TOR and PKA pathways synergize to prevent cell cycle exit and G1 arrest. (A) Flow cytometry analysis of *tor2-51*, *tor2-51 pka1Δ* and *tor2-51 cgs1Δ* strains. Cells were grown to mid-exponential phase in minimal medium at 25°C and then shifted to the restrictive temperature of 32°C to inactivate Tor2. Samples were taken at the indicated times after Tor2 inactivation. (B) *tor2-51*, *tor2-51 pka1Δ* and *tor2-51 cgs1Δ* cells were grown to mid-exponential phase at 25°C and shifted to 32°C. Samples were collected at the indicated times for RNA extraction. Northern blot was performed and hybridized with a probe against the *rum1+* gene. Ribosomal RNA (rRNA) was stained with methylene blue and used as loading control. (C) Wild-type, *pka1Δ*, *rum1d3′1* and *rum1d3′1 pka1Δ* cells were collected during mid-exponential phase for RNA extraction. Northern blot was performed and hybridized with a probe against *rum1*+. rRNA stained with methylene blue was used as loading control.

We combined the temperature-sensitive allele of the *tor2* gene, *tor2-51*, with deletions of the catalytic subunit of PKA (*pka1Δ*) or the regulatory subunit (*cgs1Δ*) and measured the DNA content by flow cytometry analysis. As shown in Fig. 1A, *tor2-51* control cells began to accumulate with 1C DNA content about 8 hours after Tor2 inactivation. The double mutant *tor2-51 pka1Δ* showed a faster rate of accumulation of cells with 1C DNA content, and G1 cells were observed 4 hours after Tor2 inactivation. Finally, in the *tor2-51 cgs1Δ* double mutant no G1-arrested cells were observed 8 hours after Tor2 inactivation. These results indicate that the TOR and the PKA pathways cooperate to prevent cell cycle exit and G1 arrest in fission yeast.

Consistent with the FACS data, the inactivation of Tor2 led to the accumulation of the G1-specific *rum1* transcript [7]. The level of *rum1* mRNA was higher in *pka1Δ* cells and lower in *cgs1Δ* cells than in the control (Fig. 1B). To determine whether the increase in *rum1* mRNA in *pka1Δ* cells was due to transcription activation or mRNA stabilization, we used a *rum1* allele lacking the AU-rich elements in its 3′UTR, called *rum1d3′1* [27]. In this mutant, the levels of *rum1* mRNA also increased after tor2 inactivation, indicating that PKA activity was repressing *rum1* transcription (Fig. 1C).

### TOR and PKA pathways synergize to repress sexual differentiation

**Figure 2:**
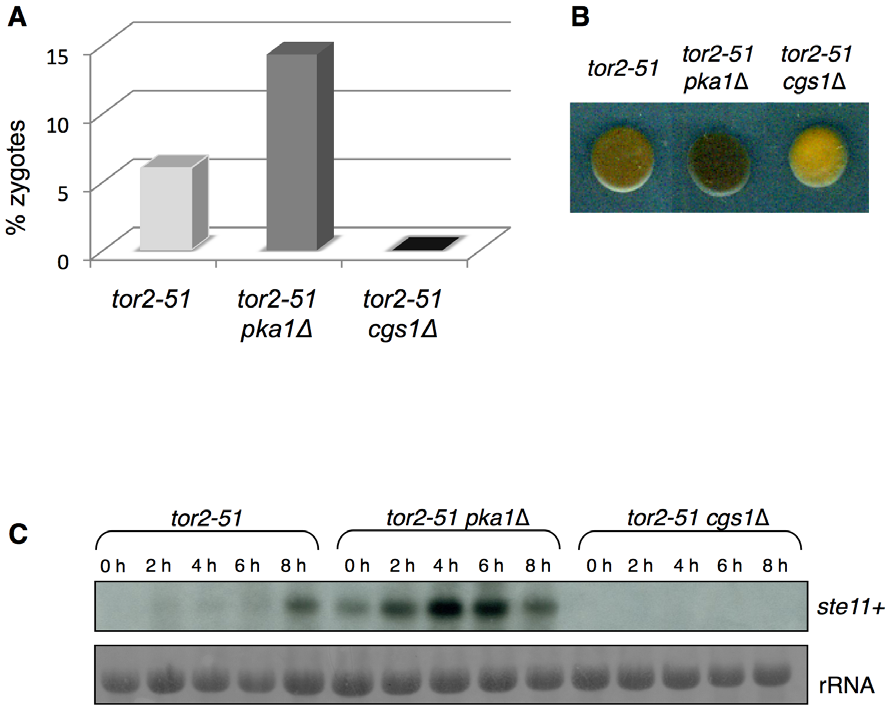
Sexual differentiation in PKA mutants after Tor2 inactivation. (A and B) *h^90^ tor2-51*, *h^90^ tor2-51 pka1Δ* and *h^90^ tor2-51 cgs1*Δ cells were incubated for 48 hours at 32°C in the presence of nitrogen (YES medium). The percentage of zygotes was determined and represented as a plot (A) and spores were stained with iodine vapour (B). *h^90^ tor2-51 pka1*Δ cells showed a higher mating efficiency than *h^90^ tor2-51* cells after Tor2 inactivation. In contrast, *h^90^ tor2-51 cgs1*Δ cells were unable to mate after Tor2 inactivation. (C) *tor2-51*, *tor2-51 pka1*Δ and *tor2-51 cgs1*Δ cells were grown to mid-exponential phase in minimal medium, washed several times and incubated in minimal medium lacking nitrogen. Samples were collected at the indicated times to extract RNA and Northern blot was performed and hybridized with a probe against the *ste11+* gene. rRNA stained with methylene blue was used as a loading control. In *tor2-51 pka1*Δ cells, *ste11* expression levels increased earlier and to a higher level than in *tor2-51*. In the *tor2-51 cgs1Δ* double mutant there was no relevant activation of *ste11*+ transcription after Tor2 inactivation.

In order to test the relative importance of the TOR and PKA pathways in repressing mating, we analyzed the mating efficiency of homothallic *h^90^ S. pombe* cells after Tor2 inactivation in wild-type, *pka1Δ* and *cgs1Δ* backgrounds. As shown in Fig. 2A and 2B, mating efficiency was higher in *tor2-51 pka1Δ* than in *tor2.51* and was almost completely abolished in *tor2.51 cgs1Δ* cells. Therefore, it seems that the TOR and PKA pathways synergize to repress sexual differentiation. In addition, Tor2 inactivation *per se* was insufficient to promote mating when the PKA activity was up-regulated.

As described previously, inactivation of either Tor2 or mutants in *pka1* promoted sexual differentiation by induction of *ste11+* mRNA. We analyzed the levels of *ste11+* mRNA after Tor2 inactivation at different time-points after the shift of *tor2-51* to the restrictive temperature (Fig. 2C). *ste11+* mRNA increased 8 hours after Tor2 inactivation in *tor2-51*, whereas in the *tor2-51 pka1Δ ste11+* mRNA levels were already high by 2 hours after the temperature shift. In contrast, in *tor2-51 cgs1Δ* on which PKA is constitutively active, *ste11+* mRNA was not induced even at 8 hours after Tor2 inactivation. This lack of *ste11+* mRNA induction is probably the reason of the sterility of *tor2-51 cgs1Δ* cells.

### Lack of PKA activity rescues the sterility of Tor2 overexpression

**Figure 3:**
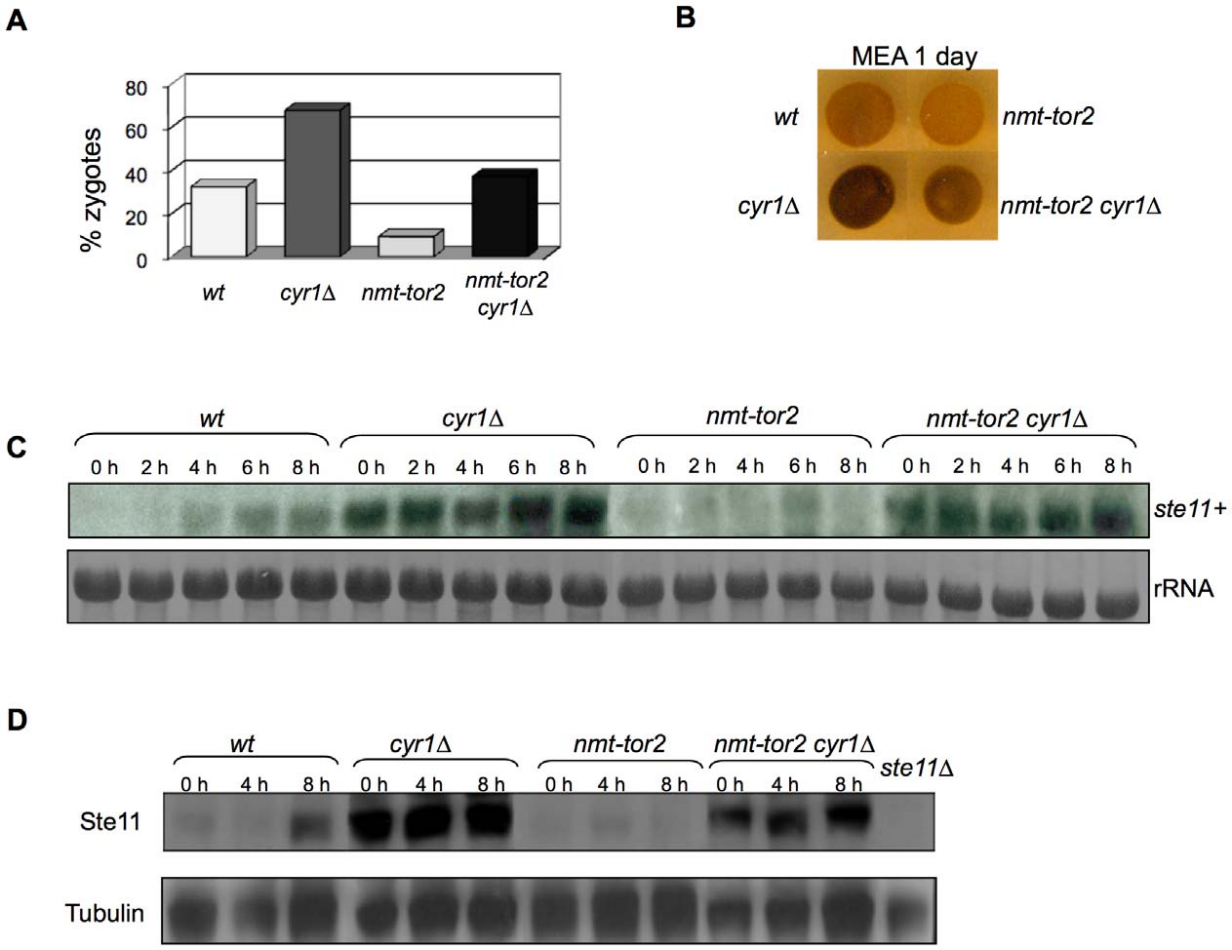
Sexual differentiation in PKA mutants overexpressing Tor2. (A and B) Cells of the opposite mating types of wild-type, *cyr1Δ*, *nmt-tor2* and *nmt-tor2 cyr1Δ* were grown to mid-exponential phase, spotted onto malt extract plates and incubated for 24 hours at 25°C. (A) Percentage of zygotes, (B) Iodine staining. Tor2 overexpression impaired sexual differentiation. This phenotype was rescued in a *cyr1Δ* background. (C and D) Wild-type, *cyr1Δ*, *nmt-tor2* and *nmt-tor2 cyr1Δ* cells were grown to mid-exponential phase in minimal medium at 25°C, washed several times and incubated in minimal medium lacking nitrogen. Cells were collected at the indicated times and RNA and protein were extracted. (C) Northern blot hybridized with a probe against *ste11+* gene. (D) Western blot using anti-Ste11 antibody. Cells overexpressing Tor2 showed lower *ste11*+ mRNA and protein levels than the wild-type. *ste11* mRNA and protein levels are clearly increased in cells lacking Cyr1 (*nmt-tor2 cyr1Δ*). rRNA and tubulin were used as loading controls.

Tor2 overexpression inhibits sexual differentiation and mating [7]. Under nitrogen depletion, cells overexpressing Tor2 show reduced *ste11+* mRNA induction and its target gene *mei3+* mRNA levels are not detectable, suggesting that Tor2 overexpression is impairing Ste11 function [7]. We therefore overexpressed Tor2 in a *cyr1Δ* mutant, lacking adenylate cyclase and hence defective in PKA activation, to determine whether the inactivation of PKA would promote sexual differentiation when Tor2 was overexpressed. We measured sexual differentiation by counting the percentage of zygotes and by staining the spores formed after 24 hours under nitrogen starvation with iodine vapors. As shown in Fig. 3A and 3B, inactivation of PKA (*cyr1Δ*) promoted mating more efficiently than wild-type cells. Tor2 overexpression in the *cyr1Δ* mutant (*nmt-tor2 cyr1Δ*) showed a phenotype between that of the wild-type and that of *cyr1Δ* cells. This result indicates that PKA inactivation is able to promote sexual differentiation even in cells in which Tor2 is overexpressed.

The Ste11 transcription factor activates the expression of many genes that are essential for mating in fission yeast, including itself [20], [28]. We analyzed the induction of *ste11+* mRNA in cells overexpressing Tor2 after nitrogen starvation (Fig. 3C). No clear induction of *ste11+* mRNA was observed in *nmt-tor2* cells. However, *ste11+* mRNA was induced to an even higher level than in the wild-type in *nmt-tor2 cyr1Δ* cells. We also observed high levels of Ste11 protein when PKA was inactive (*cyr1Δ*), regardless of the Tor2 expression levels (Fig. 3D). Thus, the PKA pathway seems to control sexual differentiation independently of the Tor2 pathway, playing a more important role in repressing *ste11+* transcription.

### PKA controls Rst2 nuclear localization under nitrogen deprivation and after Tor2 inactivation

Rst2 is a transcription factor that is required to activate *ste11+* transcription [20]. PKA negatively regulates Rst2 in rich medium by promoting its exclusion from the nucleus [21], thus repressing *ste11+* expression.

**Figure 4:**
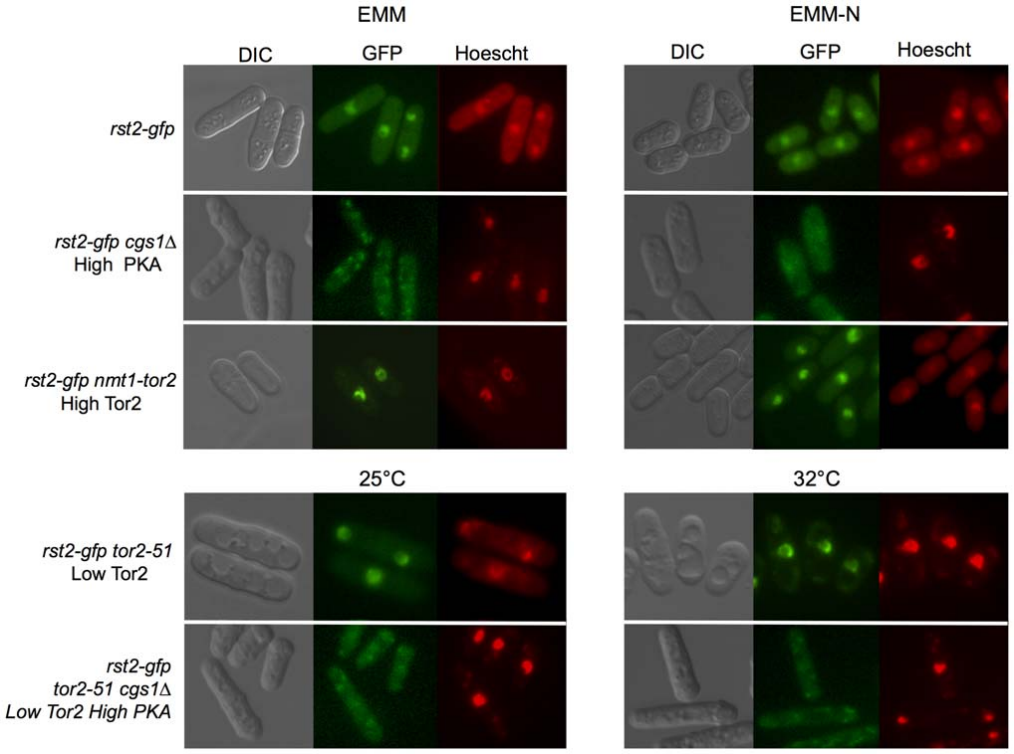
Rst2 subcellular localization is not regulated by Tor2. *rst2-gfp, rst2-gfp cgs1Δ*, *rst2-gfp nmt-tor2* cells were grown to mid-exponential phase in minimal medium and shifted to minimal medium lacking nitrogen for 4 hours. In all cases, Rst2-GFP was located in the nucleus, except in the *cgs1Δ* mutant. *rst2-gfp tor2-51* and *rst2-gfp tor2-51 cgs1Δ* cells were grown to mid-exponential phase in minimal medium at 25°C and then shifted to 32°C to inactivate Tor2. Nuclei were stained with Hoescht.

Rst2 protein levels did not change when cells were grown in medium withouth nitrogen or after Tor2 inactivation (Figure S1A, supplementary material). We analyzed the subcellular localization of Rst2 in mutants with high PKA (*cgs1Δ*) or high TOR activity (*nmt1-tor2*). In mutants with high PKA activity Rst2 was always cytoplasmic, regardless of the growth conditions (Fig. 4). This pattern of Rst2 localization was maintained in cells with low or high Tor2 activity, indicating that Tor2 does not regulate Rst2 subcellular localization.

### The PKA and TOR pathways regulate Ste11 localization

Ste11 shuttles between the nucleus and the cytoplasm. In growing cells, Ste11 is present at low levels and is pancellular. Mating pheromones and nutrient starvation trigger nuclear accumulation and increase the expression of Ste11 and its target genes [29].

**Figure 5:**
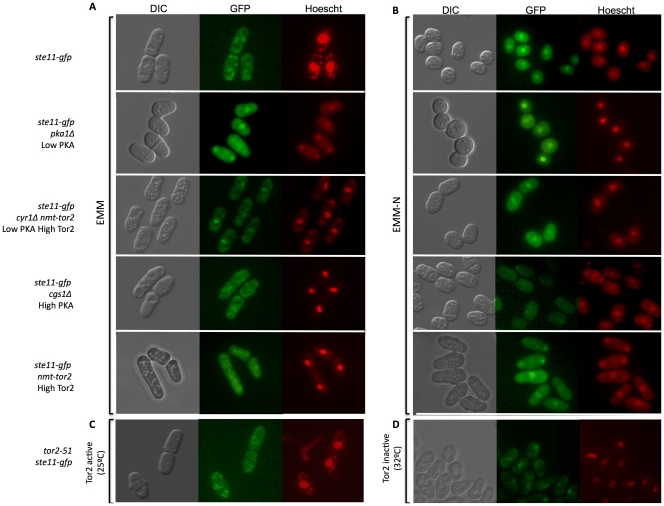
Ste11-GFP subcellular localization is regulated by PKA and TOR pathways. (A) Ste11-GFP was observed with fluorescence microscopy in wild-type (*ste11-gfp*), *ste11-gfp pka1Δ, ste11-gfp cyr1Δ nmt-tor2, ste11-gfp cgs1Δ* and *ste11-gfp nmt-tor2* cells grown in minimal medium at 32°C, and (B) after nitrogen starvation for 4 hours at 25°C. DNA was stained with Hoescht. (C and D) Ste11-GFP was also observed in *tor2-51 ste11-gfp* cells grown to mid-exponential phase at 25°C (C) and then shifted to the 32°C for 6 hours to inactivate Tor2 (D).

To determine the effects of Tor2 and PKA activities on Ste11 localization, we tagged the chromosome copy of Ste11 with GFP in the wild-type, in cells lacking PKA *(pka1Δ*), in cells expressing a constitutively active version of PKA *(cgs1Δ*), in cells overexpressing Tor2 (*nmt-tor2*), and in the *tor2-51* mutant. Exponentially growing wild-type cells showed a pancellular localization of Ste11-GFP (Fig. 5A). In cells lacking PKA, Ste11-GFP accumulated in the nucleus, even when Tor2 was overexpressed (Fig. 5A). Therefore, PKA activity inhibits Ste11 nuclear accumulation, regardless of Tor2 activity.

Under nitrogen deprivation, Ste11-GFP accumulated in the nucleus (Fig. 5B). In cells where PKA is constitutively active (*cgs1Δ*), Ste11 levels were very low and no nuclear accumulation was observed, even under nitrogen deprivation. When the PKA pathway was inactive (*pka1Δ*), Ste11 was located in the nucleus in rich medium. After Tor2 inactivation, Ste11 also accumulated in the nucleus, even in rich medium (active PKA) (Fig. 5C). When Ste11 is located in nucleus, Ste11 levels increase in the cells (Fig. S1B, supplementary material). Accordingly, both the PKA and the Tor2 pathways inhibit sexual differentiation by preventing Ste11 nuclear accumulation.

### Ste11-dependent gene expression is controlled by the TOR and PKA pathways

The above observations suggested that the TOR and PKA pathways were inhibiting Ste11 activity by excluding it from nucleus. Ste11 is a transcription factor that induces the expression of *ste11+* itself, the mating type genes, and the master regulator of meiosis, Mei2 [20], [28]. To confirm that the PKA and TOR pathways were indeed inhibiting Ste11 activity, we performed microarray analyses in cells growing in rich medium after Tor2 inactivation. Under these conditions, *ste11* and many genes involved in mating and meiosis are induced, mimicking the nitrogen starvation response [9]. In addition, we analyzed the gene expression profile in cells overexpressing Tor2 after nitrogen starvation. Table 1 lists the genes that were induced owing to the loss of Tor2 and not induced in cells overexpressing Tor2 after nitrogen starvation. Most of these genes are Ste11 targets [9]. We therefore conclude that Tor2 inhibits mating and meiosis by repressing the Ste11 transcription factor.

**Table 1:** Mating and meiosis genes induced by loss of Tor2 and not induced after nitrogen starvation in cells overexpressing Tor2.

Systematic name	Gene name	Biological Process (GeneDB)
SPAC13G7.13c	*msa1*	negative regulation of conjugation with cellular fusion
SPBC29B5.02c	*isp4*	conjugation with cellular fusion
SPAC1039.09	*isp5*	conjugation with cellular fusion
SPAC513.03	*mfm2*	regulation of conjugation with cellular fusion by signal transduction
SPAC25B8.13c	*isp7*	induction of conjugation upon nitrogen starvation
SPBC1711.02	*matmc_1*	conjugation with cellular fusion
SPBC23G7.09	*matmc_2*	conjugation with cellular fusion
SPAC11E3.06	*map1*	regulation of transcription, mating-type specific
SPAC4A8.04	*isp6*	conjugation with cellular fusion
SPAC1565.04c	*ste4*	regulation of conjugation with cellular fusion by signal transduction
SPBC32C12.02	*ste11*	conjugation with cellular fusion
SPBPJ4664.03	*mfm3*	regulation of conjugation with cellular fusion by signal transduction
SPAC11H11.04	*mam2*	pheromone-dependent signal transduction involved in conjugation with cellular fusion
SPAPB8E5.05	*mfm1*	conjugation with cellular fusion
SPBPJ4664.03	*mfm3*	regulation of conjugation with cellular fusion by signal transduction
SPBC25B2.02c	*mam1*	peptide pheromone export
SPBC24C6.06	*gpa1*	conjugation with cellular fusion
SPAC23E2.03c	*ste7*	conjugation with cellular fusion
SPAC27D7.03c	*mei2*	positive regulation of meiosis
SPAC22F3.12c	*rgs1*	negative regulation of signal transduction involved in conjugation with cellular fusion

As we have shown above, inactivation of the PKA pathway was able to rescue the sterility of Tor2 overexpression (Fig. 3A and 3B). We performed microarray analyses of wild-type cells and of Tor2-overexpressing cells with and without PKA activity after nitrogen starvation. As shown in Table S2 (supplementary material), inactivation of the PKA pathway increased the transcription levels of most Ste11 target genes, even in cells overexpressing Tor2. We thus surmise that the PKA pathway plays a more critical role than Tor2 in repressing Ste11-dependent transcription.

## Discussion

Control of cell growth and differentiation is a complex process governed by nutrient availability and growth factors that are sensed and transmitted by specialized signal transduction pathways. In fission yeast, the TOR and the PKA pathways activate cell growth and inactivate sexual differentiation. Nitrogen starvation triggers cell cycle arrest in G1 and induces the expression of the Ste11 transcription factor that is required for sexual differentiation. Here we show that inactivation of the TOR and PKA pathways synergize to promote the nitrogen starvation response and the activation of *ste11* transcription.

In fission yeast, inactivation of Tor2 promotes sexual differentiation, even in rich medium, by increasing *ste11* mRNA levels [7]. We analyzed the mating efficiency of cells after Tor2 inactivation in the wild-type and in cells lacking PKA and observed that when PKA was inactive, cells mated more efficiently than wild-type cells. This could be explained because *ste11* mRNA levels are higher in the *pka1Δ tor2-51* double mutant than in any of the single mutants or in the wild-type. On the other hand, when the PKA pathway was constitutively active, cells were completely sterile because there was no induction of *ste11* mRNA. Tor2 overexpression also impairs mating in cells under nitrogen starvation because there is no induction of *ste11* mRNA [7]. However, this phenotype was rescued when the PKA pathway was inactivated and in this background, cells showed higher levels of *ste11* mRNA and protein.

We also analyzed whether there was any cross-talk between the TOR and the PKA signaling pathways. The Rst2 transcription factor is negatively regulated by PKA and induces transcription of *ste11* [20], [21]. Rst2 is located in the nucleus but constitutively active PKA promotes its exclusion from the nucleus [20], [21]. We analyzed Rst2 localization after Tor2 inactivation and under nitrogen starvation when Tor2 was overexpressed. In all cases, Rst2 was normally localized, indicating that Tor2 does not control Rst2 localization. We also analyzed Rst2 phosphorylation and we observed that Rst2 was not hyperphosphorylated after Tor2 inactivation (data not shown). Therefore, it may be concluded that Tor2 does not control Ste11 transcription by repressing Rst2.

**Figure 6:**
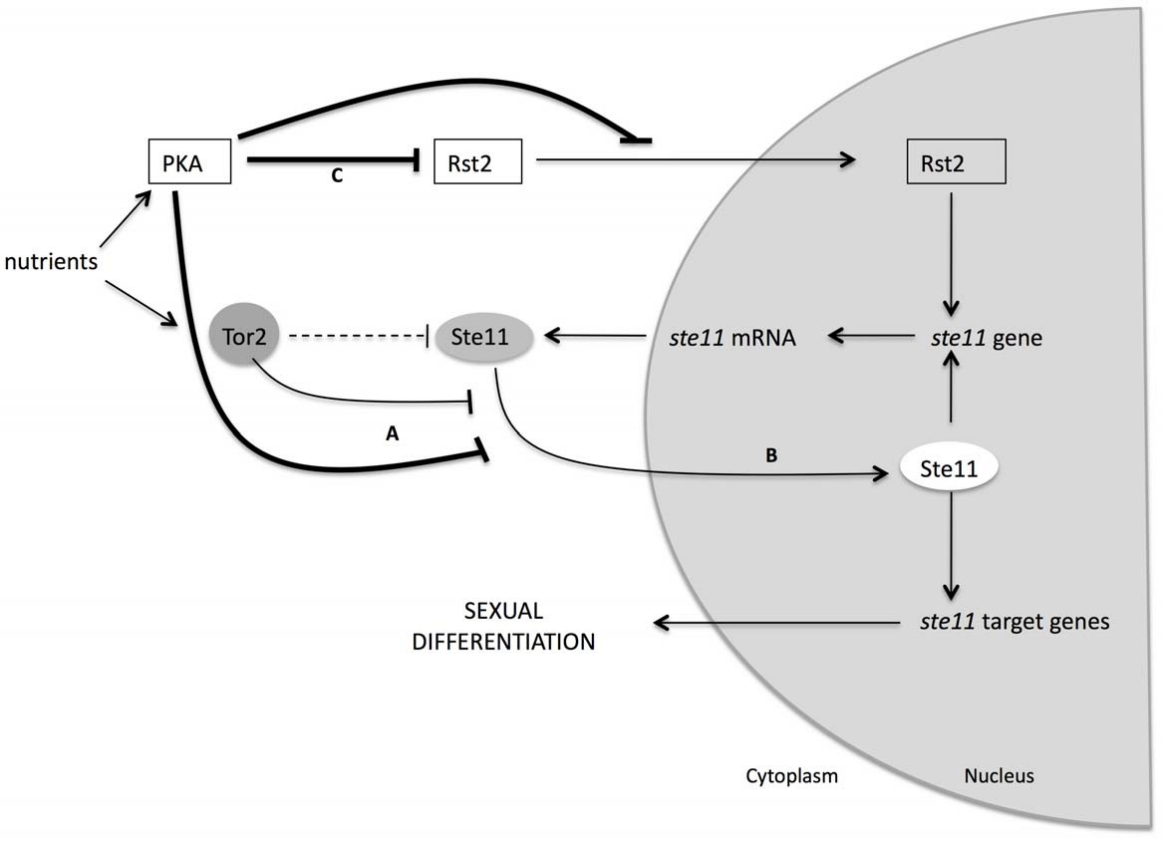
The PKA and TOR pathways regulate Ste11 subcellular localization. (A) Under good nutritional conditions, the PKA and TOR pathways inhibit Ste11 nuclear accumulation. (B) When the cells are starved for nitrogen Ste11 accumulates in the nucleus, activates the transcription of its target genes, and promotes sexual differentiation. (C) When the PKA pathway is constitutively active it represses Rst2 activity, and there is no Ste11-dependent gene expression. In this situation, sexual differentiation is repressed even if Tor2 is inactivated.

Another possibility is that the PKA and TOR signaling pathways cross-talk directly at the level of the Ste11 transcription factor. Ste11 was present at very low level and had pancellular localization in rich medium. Mating pheromones and nitrogen starvation trigger Ste11 nuclear accumulation and increase its own expression [29]. We observed that PKA inhibited Ste11 nuclear accumulation. When PKA signaling was inactive (*pka1Δ*), Ste11-GFP accumulated in the nucleus, even in rich medium. The same pattern was observed when Tor2 was inactivated, indicating that in both cases there was no nutritional starvation requirement to promote Ste11-GFP localization in nucleus. Therefore, both nutritional signaling pathways cross-talk at the level of Ste11 by regulating Ste11 localization (Fig. 6). In the case of PKA, there was another level of control: the inhibition of Ste11 expression through the Rst2 transcription factor (Fig. 6). Tor2 physically interacts with Ste11 when Tor2 is active under good nutritional conditions [7]. After Tor2 inactivation, Ste11 accumulates in the nucleus. If PKA is constitutively active, the level of Ste11 is so low (because Rst2 is inactive) that Tor2 inactivation is not sufficient to allow the accumulation Ste11 in the nucleus and the cells are unable to mate. The present study provides evidence that the subcellular distribution of Ste11 is a regulatory mechanism in which TOR and PKA pathways converge to regulate sexual differentiation in response to the nutritional environment.

## Supporting Information

Figure S1 (3.69 MB TIF)

Table S1 (0.06 MB DOC)

Table S2 (0.07 MB DOC)
