# Supplementary figures and images for: TOR and PKA Pathways Synergize at the Level of the Ste11 Transcription Factor to Prevent Mating and Meiosis in Fission Yeast
Source: PLoS One. 2010 Jul 9;5(7):e11514. doi: 10.1371/journal.pone.0011514 (PMC2901329; doi:10.1371/journal.pone.0011514)

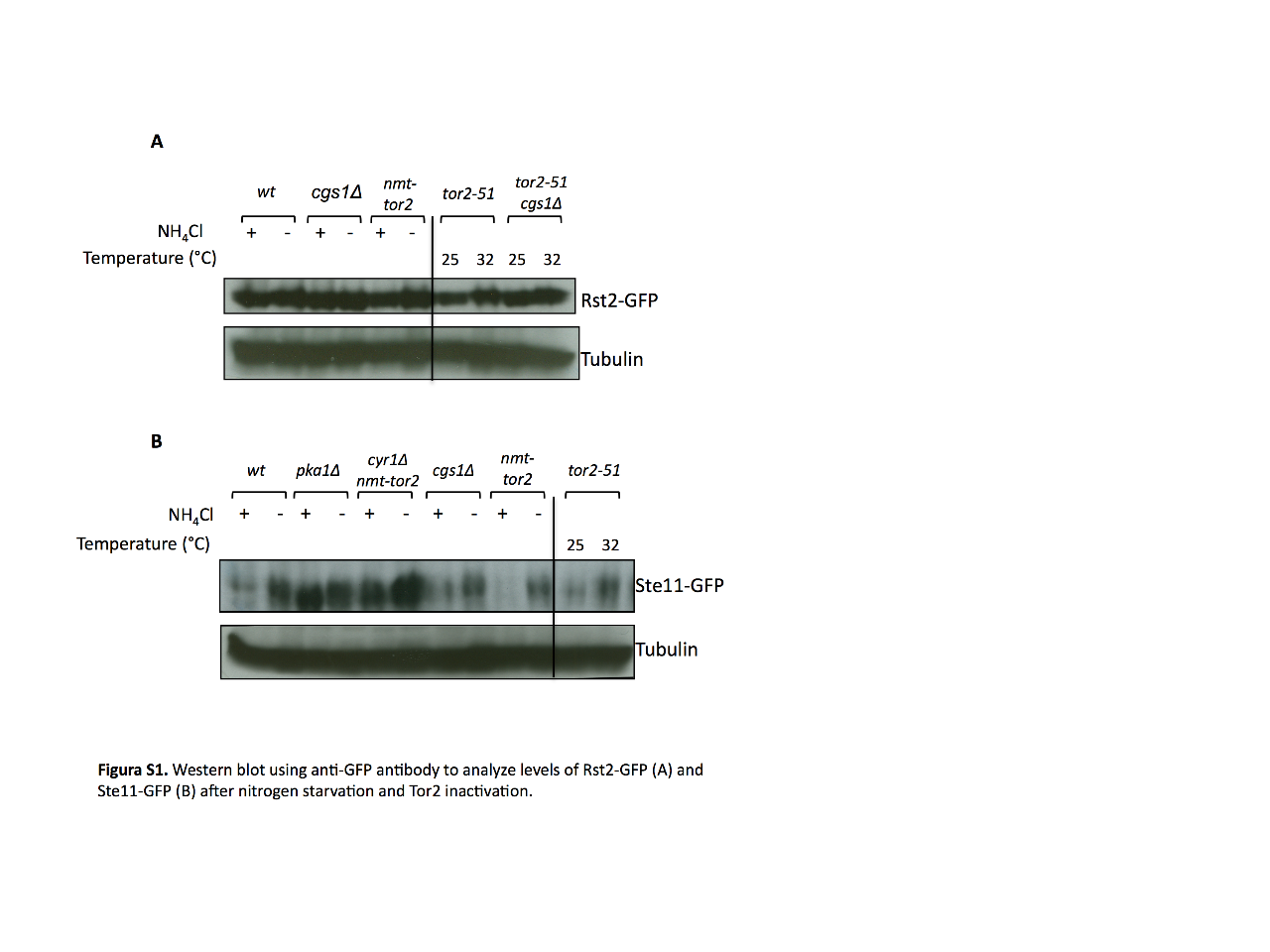

Supplement: Figure S1 — (3.69 MB TIF) [file pone.0011514.s001.tif]
