# Supplementary material for: TOR and PKA Pathways Synergize at the Level of the Ste11 Transcription Factor to Prevent Mating and Meiosis in Fission Yeast
Source: PLoS One. 2010 Jul 9;5(7):e11514. doi: 10.1371/journal.pone.0011514 (PMC2901329; doi:10.1371/journal.pone.0011514)
Supplement: Table S1 — (0.06 MB DOC) [file pone.0011514.s002.doc]

**Table S1.** Yeast strains

| Strain | Genotype | Source |
| --- | --- | --- |
| *wt* | *h- 972* | P. Nurse |
| *tor2.51* | *h- tor2-51:ura4+ ura4-d18* | Lab stock |
| *tor2.51 pka1Δ* | *h- tor2-51:ura4+ ura4-d18 pka1::ura4+* | This study |
| *tor2.51 cgs1Δ* | *h- tor2-51:ura4+ ura4-d18 cgs1::ura4+* | This study |
| *rum1d3´1* | *h- rum1-d3´1* | Lab stock |
| *rum1d3´1 pka1Δ* | *h- rum1-d3´1 pka1::ura4+* | This study |
| *pka1Δ* | *h- pka1::ura4+ ura4-d18* | M. Balasubramaniam |
| *cgs1Δ* | *h- cgs1::ura4+ ura4-d18* | M. McLeod |
| *cyr1Δ* | *h- cyr1::ura4+ ura4-d18* | Lab stock |
| *ste11Δ* | *h+ ste11::ura4+ ura4-d18* | Lab stock |
| *h^90^ tor2-51* | *h^90^ tor2-51:ura4+ ura4-d18* | Lab stock |
| *tor2-51 pka1Δ* | *h+ tor2-51:ura4+ pka1::ura4+ ura4-d18* | This study |
| *h^90^ tor2-51 pka1Δ* | *h^90^ tor2-51:ura4+ pka1::ura4+ ura4-d18* | This study |
| *tor2-51 cgs1Δ* | *h+ tor2-51:ura4+ cgs1::ura4+ ura4-d18* | This study |
| *h^90^ tor2-51 cgs1Δ* | *h^90^ tor2-51:ura4+ cgs1::ura4+ ura4-d18* | This study |
| *nmt-tor2* | *h- KanMX6:nmt1:tor2* | Lab stock |
| *nmt-tor2 cyr1Δ* | *h- KanMX6:nmt1:tor2 cyr1::ura4+ ura4-d18* | This study |
| *rst2-gfp* | *h- rst2-gfp:KanMX6* | This study |
| *rst2-gfp cgs1Δ* | *h- rst2:gfp:KanMX6 cgs1::ura4+ ura4-d18* | This study |
| *rst2-gfp nmt-tor2* | *h- rst2-gfp:KanMX6 KanMX6:nmt1:tor2* | This study |
| *rst2-gfp tor2-51* | *h- rst2-gfp:KanMX6 tor2-51:ura4+ ura4-d18* | This study |
| *rst2-gfp tor2-51 cgs1Δ* | *h- rst2-gfp:KanMX6 tor2-51:ura4+ cgs1::ura4+ ura4-d18* | This study |
| *rst2-HA* | *h- rst2-HA:KanMX6* | This study |
| *rst2-HA tor2-51* | *h- rst2-HA:KanMX6 tor2-51:ura4+ ura4-d18* | This study |
| *ste11-gfp* | *h- ste11-gfp:KanMX6* | This study |
| *ste11-gfp cgs1Δ* | *h- ste11-gfp:KanMX6 cgs1::ura4+ ura4-d18* | This study |
| *ste11-gfp pka1Δ* | *h- ste11-gfp:KanMX6 pka1::ura4+ ura4-d18* | This study |
| *ste11-gfp nmt-tor2* | *h- ste11-gfp:KanMX6 KanMX6:nmt1:tor2* | This study |
| *ste11-gfp cyr1Δ nmt-tor2* | *h- ste11-gfp cyr1::ura4+ KanMX6:nmt1:tor2 ura4-d18* | This study |
| *ste11-gfp tor2-51* | *h- ste11-gfp:KanMX6 tor2-51:ura4+ ura4-d18* | This study |
