# Supplementary material for: TOR and PKA Pathways Synergize at the Level of the Ste11 Transcription Factor to Prevent Mating and Meiosis in Fission Yeast
Source: PLoS One. 2010 Jul 9;5(7):e11514. doi: 10.1371/journal.pone.0011514 (PMC2901329; doi:10.1371/journal.pone.0011514)
Supplement: Table S2 — (0.07 MB DOC) [file pone.0011514.s003.doc]

**Table S2.** Ste11 target genes not expressed in cells overexpressing Tor2 and expressed in cells overexpressing Tor2 in *cyr1Δ* mutant background.

| **Systematic name** | **Gene Name** | **Biological Process (GeneDB)** |
| --- | --- | --- |
|  |  |  |
| **Regulation of meiosis** | | |
| SPAC27D7.03c | *mei2* | positive regulation of meiosis |
| SPNCRNA.103 | *sme2* | meiosis |
| SPBC19C2.05 | *pat1* | negative regulation of conjugation with cellular fusion |
| SPBC2D10.06 | *rep1* | regulation of transcription, meiotic |
| SPAC15A10.03c | *rad54* | meiotic gene conversion |
|  |  |  |
| **Regulation of mating/pheromone response** | | |
| SPAC31G5.09c | *spk1* | regulation of conjugation with cellular fusion by signal transduction |
| SPAC22F3.12c | *rgs1* | negative regulation of signal transduction involved in conjugation with cellular fusion |
| SPCC1442.01 | *ste6* | conjugation with cellular fusion |
| SPAC23E2.03c | *ste7* | conjugation with cellular fusion |
| SPBC24C6.06 | *gpa1* | conjugation with cellular fusion |
| SPBC32C12.02 | *ste11* | conjugation with cellular fusion |
| SPAC11E3.06 | *map1* | regulation of transcription, mating-type specific |
| SPAC1565.04c | *ste4* | regulation of conjugation with cellular fusion by signal transduction |
|  |  |  |
| **Cell cycle regulators** | | |
| SPBC32F12.09 | *rum1* | cell cycle arrest in response to nitrogen starvation |
| SPBP23A10.04 | *apc2* | anaphase-promoting complex-dependent proteasomal ubiquitin-dependent protein catabolic process |
| SPAPB1A10.02 | *scm3* | cell cycle |
|  |  |  |
| **Wall and Spore formation** | | |
| SPAC1F5.08c | *ehs1* | regulation of fungal-type cell wall biogenesis |
| SPAC27D7.04 | *omt2* | ascospore formation |
|  |  |  |
| **Signal transduction** | | |
| SPAC1F5.09c | *shk2* | MAPKKK cascade involved in conjugation with cellular fusion |
| SPCC162.10 | *ppk33* | signal transduction |
| SPAC26F1.04c | *etr1* | cellular response to stress |
| SPCC338.18 |  | cellular response to stress |
| SPAC8C9.16c | *mug63* | cellular response to oxidative stress |
| SPBC1604.01 | *mug158* | cellular response to stress |
|  |  |  |
| **Mating type-specific genes from fission yeast (M specific)** | | |
| SPAPB8E5.05 | *mfm1* | conjugation with cellular fusion |
| SPAC513.03 | *mfm2* | regulation of conjugation with cellular fusion by signal transduction |
| SPBPJ4664.03 | *mfm3* | regulation of conjugation with cellular fusion by signal transduction |
| SPBC25B2.02c | *mam1* | peptide pheromone export |
| SPAC11H11.04 | *mam2* | pheromone-dependent signal transduction involved in conjugation with cellular fusion |
| SPAP11E10.02c | *mam3* | agglutination involved in conjugation with cellular fusion |
| SPAC10F6.12c | *mam4* | response to pheromone during conjugation with cellular fusion |
| SPAC1296.03c | *sxa2* | conjugation with cellular fusion |
| SPAPB1A10.04c | *cwp1* | protein amino acid geranylgeranylation |
|  |  |  |
| **Others** | | |
| SPAC18G6.01c |  | unknown function |
